# Significance of pyrolytic temperature, application rate and incubation period of biochar in improving hydro-physical properties of calcareous sandy loam soil

**DOI:** 10.1038/s41598-024-57755-y

**Published:** 2024-03-25

**Authors:** Ammar A. Albalasmeh, Mohammad Z. Quzaih, Mamoun A. Gharaibeh, Munir Rusan, Osama E. Mohawesh, Samer R. Rababah, Ahmad Alqudah, Abdulaziz G. Alghamdi, Amir Naserin

**Affiliations:** 1 Department of Natural Resources and Environment, Faculty of Agriculture, Jordan University of Science and Technology, P.O. Box 3030, Irbid, 22110 Jordan; 2 Department of Plant Production, Faculty of Agriculture, Mutah University, P.O. Box: 7, Karak, 61710 Jordan; 3 Department of Civil Engineering, Faculty of Engineering, Jordan University of Science and Technology, P.O. Box 3030, Irbid, 22110 Jordan; 4 Department of Biological and Environmental Sciences, College of Art and Science, Qatar University, 2713 Doha, Qatar; 5 Department of Soil Sciences, College of Food and Agricultural Science, King Saud University, PO Box 2460, 11451 Riyadh, Saudi Arabia; 6 Department of Water Engineering, Agricultural Sciences and Natural Resources University of Khuzestan, Mollasani, 6341773637 Iran

**Keywords:** Incubation, Olive pomace biochar, Soil hydraulic properties, Soil amendment, Soil structure, Environmental sciences, Hydrology

## Abstract

Biochar is increasingly recognized for its ability to enhance hydro-physical properties of soil, offering promising solutions for improving soil structure, water retention, and overall agricultural productivity. In this study, sandy loam soil was amended at different rates (0, 15, 30, and 60 t ha^−1^) of biochar produced from olive pomace (Jift) at different pyrolysis temperatures (300, 400, 500, and 600 °C), and incubated for 30, 60, and 90 days. The biochar-amended soils were collected for analysis after each incubation period for infiltration rate, aggregate stability, soil water retention, water repellency, and penetration resistance. At 300 °C, aggregate stability increased with biochar amendments; the highest value (65%) was after 60 days of incubation. At other pyrolysis temperatures, aggregate stability decreased, or no effect of temperature was observed. Also, at 300 °C, the infiltration rate was decreased with biochar application and the lowest value of (0.14 ml/min) was at 90 days of incubation. At other pyrolysis temperatures, the infiltration rate was increased with increased biochar application rate. Water retention was increased with biochar application at 300 °C; however, biochar application did not affect water retention at other pyrolysis temperatures. These results strongly suggest the improvement of soil physical and hydraulic properties following the addition of biochar amendment. Overall, biochar had positive effects on hydro-physical properties. The biochar produced at 300 °C pyrolysis temperature was the most beneficial to agriculturally relevant hydraulic conditions. However, field assessments are necessary to evaluate the long-term effects of biochar on hydro-physical properties.

## Introduction

The global intensification of agricultural production is required to ensure food security for a growing global population. However, in arid and semiarid regions where the soil is coarse-textured soil, low water- and nutrient-holding capacities impose significant limitations on sustainable agriculture^1,2^. Therefore, to maximize agricultural production, scientists search for amendments that can improve the hydro-physical properties of these soils. Biochar produced from organic wastes pyrolysis has been used as a soil amendment to ameliorate soil physicochemical and hydrological properties^3–5^.

Terra Preta is a proven example of the effect of biochar as a soil amendment to improve soil quality^6^. Biochar is defined as a highly porous carbon-rich byproduct of the pyrolysis of organic wastes in the absence or limited presence of oxygen. The physicochemical properties of the produced biochar depend on the pyrolysis conditions (temperature and duration) and the feedstock sources (agro-forestry biomass, organic waste materials, sludge, and animal manure)^7–9^. Recent research demonstrated that incorporating biochar into soil improves the soil's ability to retain water and nutrients and reduces nutrient losses via leaching^10–12^. Several previous studies indicated the effects of biochar on the soil’s properties largely depended on soil type, feedstock materials, and biochar dosage. Zhang et al.^13^ found that adding a 2% application rate of timber-harvesting residues biochar increased plant-available water, particularly for the silt loam soil, and the particle size of the used biochar had no effect on plant-available water. Yang and Lu^14^ concluded that biochar at 22.5 t ha^−1^ application rate increased the plant-available water by 15.2% to 42.4%, depending on the feedstock used to produce the biochar. Ibrahim and Horton^15^ found that adding date palm biochar increases plant-available water and decreases hydraulic conductivity, where the maximum results were obtained at a 4% application rate. Similarly, Alkhasha et al.^16^ reported that date palm biochar applied at a rate of 2% increased soil water retention by 68%, lowered hydraulic conductivity and infiltration rate, and improved crop productivity in sandy soils. Moreover, Ibrahim et al.^17^ concluded that cumulative evaporation, saturated hydraulic conductivity, and infiltration rate decreased by increasing the application rates of biochar produced from Conocarpus while at the same time enhancing the soil water retention and aggregate stability. These enhancements improve soil quality, increase water use efficiency, and reduce water stress on crop yields. Alghamdi et al.^4,5^ reported a significant decrease in soil-saturated hydraulic conductivity for all treatments compared to control after adding biochar produced at different pyrolysis temperatures and application rates.

Notably, soil physical and hydraulic properties change strongly correlate with the amount of applied biochar. Most research on biochar’s effect on soil physical qualities used high biochar application rates of one to two hundred tons per hectare, which is impractical for actual farming^14,18^. Some research produced contradictory results and focused on only a tiny subset of physical and hydraulic parameters. Therefore, investigating biochar’s effects on various physical and hydraulic soil parameters is crucial^19–21^. Furthermore, the influence of pyrolysis temperature is rarely considered in recent investigations of the hydraulic characteristics of biochar-amended soil. More study is required to identify modifications induced by low-dosage biochar. Furthermore, applying biochar in small quantities, not only lowering production costs for farmers but also it enhances its potential for contributing to environmental protection. However, there has been a lack of research into the correlations between biochar pyrolysis temperature, application rate, incubation period, and the resulting changes in hydraulic, physical, and chemical properties. Investigating the intricate interplay between pyrolytic temperature, application rate, and incubation period of biochar represents a pioneering endeavor in soil science. Our research unveils the profound significance of these factors in enhancing the hydro-physical properties of calcareous sandy loam soil. Therefore, the objective of this study is to evaluate biochar's impact produced under different pyrolysis temperature, applied at different application rate and incubated for different incubation time on soil's physical, hydrological, and chemical properties.

## Methodology

### Biochar production and characterizations

Olive pomace (locally known as Jift) was obtained from a local Olive mill and used as feedstock for biochar production. Jift samples were subjected to different pyrolysis temperatures (300, 400, 500, and 600 °C) using a muffle furnace for 1 h without an oxygen supply. After pyrolysis, biochar was ground and passed through a 0.25 mm sieve.

Electrical conductivity (EC) was measured using an EC meter (Orion Star A212, Thermo Scientific) in a 1:20 (biochar: water) solution^9^. pH was measured using a pH meter (Orion Star A211, Thermo Scientific) using boiling water at a ratio of 1:10 (biochar: water) solution^22^. The ash content of biochar was measured following the ASTM D2866-11 method^23^. Briefly, 1 g biochar was combusted in a muffle furnace for 6 h at 650 °C, cooled down in a desiccator, and then ash content was measured by sample mass differences.

### Soil sampling and characterizations

Samples of sandy loam soil were collected from the soil surface (0–20 cm) from Al-Mafraq province northeast of Jordan. The soil samples were air-dried and passed through a 2 mm sieve. The soil’s physical and chemical properties were measured using standard methods as presented in Table 1. Soil texture by hydrometer method^24^, bulk density using the core technique^25^, organic matter (OM) using the wet oxidation method^26^, pH and electrical conductivity (EC) using 1:1 (soil: water) following Mclean^27^ and Rhoades^28^, respectively.

**Table 1:** Physical and chemical properties of the soil.

Parameter	Unit	Value
Soil texture	–	Sandy loam
Sand	%	58
Silt	%	32
Clay	%	10
Bulk density	g cm^−3^	1.3
pH	–	7.7
EC	dS m^−1^	1.1
OM	%	2.4

*EC* electrical conductivity, *OM* organic matter.

### Incubation experiment

Soil samples were mixed with biochar produced at different pyrolysis temperatures (300, 400, 500, and 600 °C) at different application rates (0-, 15-, 30-, and 60-ton ha^−1^). Open-top plastic columns (11 cm internal diameter × 7 cm long) were used in the experiment. A filter paper was placed on the bottom of each column; a homogenized soil-biochar mixture was then packed gently into each column to obtain an equivalent bulk density of the field. The biochar treatments were: control (0), 15, 30, and 60-ton ha^−1^. Each treatment was replicated three times, and the experimental columns were arranged in a completely randomized design (CRD). During the 90-day incubation period, the soil moisture was maintained at field capacity by adjusting the water content every 3 days (weight-based). The biochar-amended soils were collected for physical and chemical analysis after 30, 60, and 90 days of incubation.

### Soil properties measurements

#### Infiltration rate

Infiltration rate (IR) was measured using a minidisk infiltrometer (Decagon Devices, Inc., Pullman, WA, USA). The minidisk infiltrometer (MDI) was filled with water, and the suction was adjusted by the suction control tube to 5 cm as an optimal suction setting^29^. After that, the MDI was placed on the soil column, and infiltrated water volume was recorded every 30 s until steady-state conditions were reached. Cumulative infiltration and time can be fitted with the function:

$$I=S\sqrt{t}+At,$$

where I [cm] is the cumulative infiltration, t [s] is the cumulative time, S [cm s^−1/2^] is soil sorptivity, and A [cm/s] is proportional to saturated hydraulic conductivity.

#### Aggregate stability

Soil aggregate stability (AS) was measured using the wet sieving method^30^. 4 g of air-dried soil was weighed, transferred to 4 cm diameter sieves with 250 µm mesh size, and pre-wetted by capillary from the bottom using a wet paper towel placed underneath the mesh to minimize air slaking. Afterward, the sieves were submerged in labeled cans filled with distilled water and shaken using a wet sieving apparatus (Eijkelkamp Agrisearch Equipment, Giesbeek, Netherlands) at a regular up-and-down motion for three minutes. The mass of soil collected in the cans passing the sieves (*M*_1_) was then determined after evaporating the supernatant water in the oven. The aggregates remaining above the sieves were subjected to a second round of wet sieving using another set of labeled cans filled with dispersing solution (2 g L^−1^ sodium hexametaphosphate). The mass of soil collected in the second set of cans (*M*_2_) was determined by evaporating the supernatant solution in the oven, and the percentage of the water-stable aggregates was calculated as

$$A.S \%=\left(\frac{{M}_{2}-0.2}{{M}_{1}+ {M}_{2}-0.2}\right)\times 100.$$

#### Soil water retention

A pressure plate apparatus was used to determine the soil’s water retention^31^. Subsamples were placed in cylindrical cores. Samples were saturated with water by capillary. After that, the pressure chamber was closed tightly, and the pressure gauge was placed at different pressures (0.1, 0.3, 0.5, 1, 3, 5, 10, and 15 bars). Each pressure value was allowed to reach equilibrium, and then all cores were removed, weighed, oven-dried at 105 °C for 24 h, and re-weighed after drying to calculate the corresponding water content. Available water (AW) content was calculated as follows:

$$AW=FC-PWP,$$

where FC is water content at field capacity (0.1 bar), and PWP permanent is water content at wilting point (15 bar).

#### Water repellency

Soil water repellency was assessed using the water drop penetration time (WDPT) test method^32^. In brief, a droplet of water was placed by a dropper on the surface of the tested soil, and the time required for the droplet to infiltrate was recorded. The class of hydrophobicity was determined using Dekker et al.^33^ classification (Table 2).

**Table 2:** Water repellency categories corresponding to the water drop penetration times (WDPT).

WDPT (s)	Repellency category
≤ 5	Non-repellent
5–60	Slightly repellent
60–600	Strongly repellent
600–3600	Severely repellent
≥ 3600	Extremely repellent

#### Penetration resistance

Penetration resistance was determined by a pocket penetrometer (HUMBOLDT, Mfg. Co, USA). The pocket penetrometer was placed into the soil surface and pushed it into the soil until its 6.4 mm diameter round tip was entered into the soil. Then, the permanent scale on the piston barrel read approximate unconfined compressive strength in kg cm^−2^.

#### Organic matter content

Soil organic matter (SOM) was measured according to Walkley and Black method^26^. This method involves the reduction of potassium dichromate (K_2_Cr_2_O_7_) by organic compounds and subsequent determination of the unreduced dichromate by an oxidation–reduction titration with ferrous sulfate.

#### Electrical conductivity and pH

Soil electrical conductivity and pH were measured in 1:1 extract (soil: water) following the method described by Rhoades^28^ and Mclean^27^, respectively. 30g of each soil was placed in a centrifuge tube, 30 ml of distilled water was added, placed on a shaker, shaken for two hours, and centrifuged to extract the solution from the soil. The solution was then filtrated using filter paper. After that, the EC and pH for the extracted solution were measured using an EC meter (Orion Star A212, Thermo Scientific) and a pH meter (Orion Star A211, Thermo Scientific), respectively.

### Statistical analysis

Data analysis was conducted using the statistical analysis software program R. Significant differences among means were analyzed by Tukey’s HSD (honestly significant difference) test at a significance level α < 0.05. All measurements in this study were conducted on three replicate samples. All reported data points denote the means of the replicates.

## Results and discussion

### Effect of pyrolysis temperature on selected properties of biochar

The properties of the produced biochar affected by pyrolysis temperature are tabulated in Table 3. The result shows a decrease in the yield of the produced biochar as the pyrolysis temperature increases. It decreased from 52.44 to 27.78% at 300 and 600 °C, respectively. This decrease could be explained by the thermal breakdown of the jift component and the loss of volatile matter and moisture^9,34,35^. pH increased with increasing pyrolysis temperature from 8.31 to 9.54 at 300 and 600 °C, respectively. Similarly, EC increased with increasing pyrolysis temperature from 0.81 to 5.26 dS m^−1^ at 300 and 600 °C, respectively. These results are well documented and in agreement with the published literature such as Pariyar et al.^36^. The pH value of the produced biochar from different feedstocks tends to be alkaline (pH > 7). This could be because of the accumulated alkali salts in the produced biochar and the increase of inorganic carbonates with increasing pyrolysis temperatures^9,37^. The increase in the EC value could be referred to the increase in the salts in the produced biochar with increasing pyrolysis temperature^38,39^. Moreover, Table 3 shows that as pyrolysis temperature increased, the ash content increased, and the volatile matter content decreased. Ash content increased from 7.45 to 13.63%, and volatile matter decreased from 73.2% to 45.4% when the pyrolysis temperature increased from 300 to 600 °C. Many studies reported similar findings^4,9,35,38^.

**Table 3:** Effect of pyrolysis temperatures on selected properties of biochar.

Pyrolysis temperature (°C)	Yield (%)	pH	EC (dS m^−1^)	Ash content (%)	VM (%)
300	52.44	8.31	0.81	7.45	73.20
400	42.22	8.94	1.72	10.15	64.20
500	28.44	9.37	3.5	12.77	53.27
600	27.78	9.54	5.26	13.63	45.40

*EC* electrical conductivity, *VM* volatile matter.

### Effect of biochar on soil properties

#### Effect of biochar on infiltration rate

**Figure 1:**
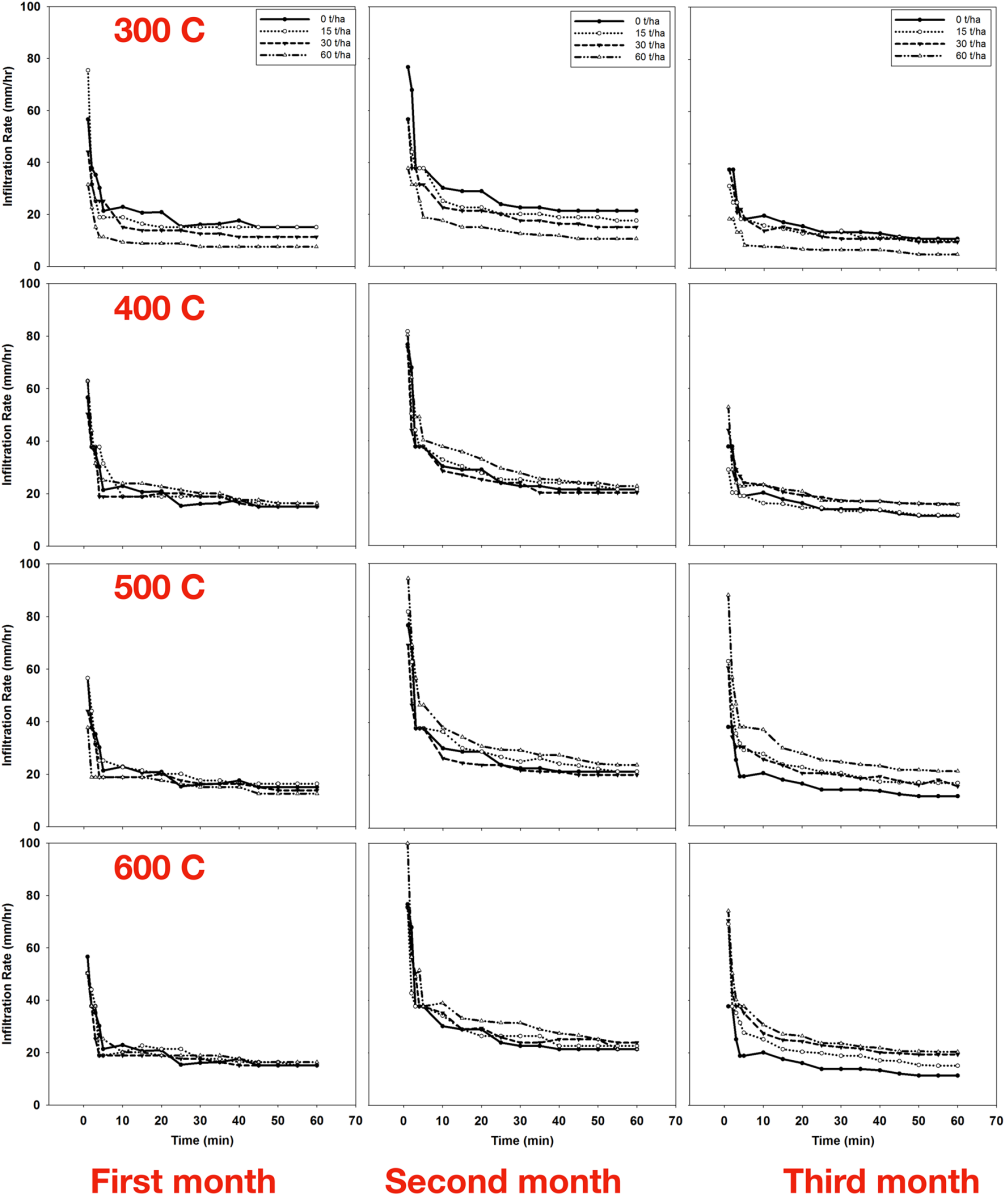
Effect of biochar that produced under different pyrolysis temperature (first row) 300 °C (second row) 400 °C, (third) 500 °C, (fourth) 600 °C and different application rates on soil infiltration rate after first—(left column), second—(middle column) and third—(right column) month of incubation.

The effect of produced biochar at different pyrolysis temperatures and applied to the soil at different application rates after the first, second, and third months of incubation on soil infiltration rates are depicted in Fig. 1. After the first month of incubation, the results showed no differences in the infiltration rate at different application rates under different pyrolysis temperate compared to control (15.1 mm h^−1^) except under 300 °C pyrolysis temperature (7.5 mm h^−1^). Similar results were obtained after 2 months of biochar incubation (second month). The only decrease in the infiltration rate was under 300 °C produced biochar treatments. The infiltration rate decreased to 17.6, 15.1, and 10.6 mm h^−1^ after applying 15, 30, and 60 ton ha^−1^, respectively.

However, after the third month of application, the results were contradicted. The infiltration rate decreased under 300 °C pyrolysis temperature treatment and increased under other pyrolysis temperatures. The infiltration rate increased from 11.3 mm h^−1^ in the control treatment to 15.1, 19.4, and 20.4 mm h^−1^ after the 15, 30, and 60-ton ha^−1^ application rates, respectively. Alghamdi et al.^4^ also found that the cumulative infiltration decreases significantly from 40.9 to 37.7 mm after incorporating biochar produced at 300 °C with soil. They attributed the decrease in soil water infiltration to the fine biochar particles that fill the pores that limit water pathways.

Table 4 represents the effect of biochar application on the final infiltration rate. The results showed that applying biochar to the soil decreased the final infiltration rate at 300 °C under all incubation periods. At other pyrolysis temperatures (400, 500, and 600 °C), the final infiltration rate had no significant effect with biochar application after the first and second months of incubation. However, it increased after the third month of incubation for 400, 500, and 600 °C pyrolysis temperatures.

**Table 4:** Effect of biochar application on soil final infiltration rate (mm hr^−1^).

Temperature (°C)	Incubation time											
	0–30 days				30–60 days				60–90 days			
	Application rate (ton ha^−1^)											
	0	15	30	60	0	15	30	60	0	15	30	60
300	15.1b	15.1b	11.3e	7.5f	21.4c	17.6e	15.1f	10.6g	11.3i	10.6j	10.0k	5.3l
400	15.1b	15.1b	15.1b	16.4a	21.4c	21.4c	20.1d	22.6b	11.3i	11.6h	15.8e	15.6f
500	15.1b	16.4a	13.8c	12.6d	21.4c	21.4c	20.1d	23.9a	11.3i	16.4d	15.1g	20.9a
600	15.1b	15.1b	15.1b	16.4a	21.4c	22.6b	23.9a	21.4c	11.3i	15.1g	19.4c	20.4b

Values within same incubation time followed by the same letter are not significantly different at the ⍶ = 0.05 probability level according to Tukey’s honestly significant difference (HSD) test.

Biochar would be expected to reduce the infiltration rate in coarse-textured soil while increasing it in fine-textured soil^40,41^. The results of this research are consistent with the data reported by Page-Dumroese et al.^42^, who showed that mixing biochar with sandy loam soil at rates 0, 1, 5, and 10 ton ha^−1^ decreased infiltration rate from 5.2 to 3.3, 1.3 and 0.8 mL min^−1^, respectively. In another study, Novak et al.^43^ showed that the application of biochar produced from pyrolysis of pine chips (Pinus taeda) at 500 °C to the sandy loam soil increased infiltration rate from 0.157 to 0.219 mL min^−1^ compared to the control (0.095 mL min^−1^). In contrast, Hardie^44^ showed that applying biochar produced from acacia whole tree green waste to the sandy loam soil does not affect the infiltration rate.

Increasing the temperature of pyrolysis reduces the size of the produced biochar particles^10^. Smaller biochar particles exhibit enhanced interaction and blending with soil particles compared to larger ones, leading to the formation of stronger soil aggregates. This is due to soil–microbe–biochar interactions^45^. Consequently, this phenomenon contributes to the enlargement of macropores, thereby enhancing the infiltration and hydraulic conductivity of loamy soils^46^.

#### Effect of biochar on aggregate stability

**Figure 2:**
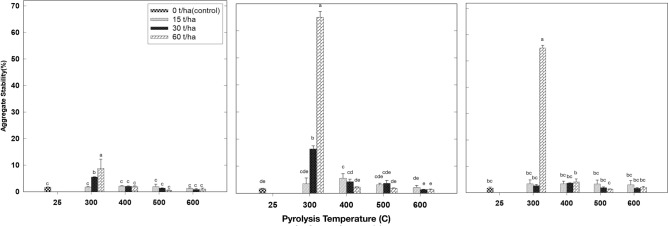
Effect of biochar that produced under different pyrolysis temperature and application rates after first (left), second (middle) and third (right) month of incubation on soil macroaggregate formation/stability. Values are averages over three replicates of the treatments, with error bar representing the standard error on y-axis. Means within same incubation time (subfigure) followed by the same letter are not significantly different at the ⍶ = 0.05 probability level according to Tukey’s honestly significant difference (HSD) test.

Figure 2 illustrates the impact of biochar produced at different pyrolysis temperatures and applied to the soil at different application rates after a 3-month incubation period on aggregate stability. The results indicate that aggregate stability increased as the biochar application rate increased, particularly when produced at a pyrolysis temperature of 300 °C and incubated for one month. In this scenario, it improved from 1.7% in the control treatment to 1.8%, 5.6%, and 8.8% with biochar application rates of 15, 30, and 60 ton ha^−1^, respectively (see Fig. 2a). Aggregate stability peaked at 65% during the second month of incubation in the treatment where biochar produced at 300 °C and applied at a rate of 60 ton ha^−1^ was used (Fig. 2b). A similar pattern was observed in the third month of incubation (Fig. 2c), with aggregate stability reaching a maximum of 54.9% when using biochar produced at 300 °C and applied at a rate of 60 tons ha^−1^. Conversely, for all treatments conducted at pyrolysis temperatures of 400 °C, 500 °C, and 600 °C, the aggregate stability remained within the range of 5% to 15% (Fig. 2). Although this increase may appear relatively modest when compared to the 300 °C pyrolysis temperature treatment, it represents a noteworthy enhancement in aggregate stability, particularly in sandy loam soil, when compared to the control.

The findings from our study align with previous research of biochar’s impact on soil aggregate stability. Brodowski et al.^47^ proposed that biochar could enhance the formation of soil aggregates and shield them from degradation by serving as a binding agent. Our results are consistent with the observations made by Ma et al.^48^, who demonstrated that applying biochar to soil led to an increase in macroaggregates. Similarly, Khademalrasoul et al.^49^ and Ouyang et al.^50^ found that soil aggregate stability improved as the biochar application rate increased. However, contrasting results have also been reported. For instance, Peng et al.^51^ found that applying biochar to the soil at lower application rates (1%) had no discernible effect on aggregate stability. Likewise, Liu et al.^52^ observed that applying biochar produced at a pyrolysis temperature of 660 °C to sandy loam soil at rates of 4, 8, and 16 g kg^−1^ did not significantly influence aggregate distribution or stability. Other studies have reported mixed or negligible effects. Jeffery et al.^53^ found that applying biochar to the soil had no significant impact on aggregate stability. Conversely, Ouyang et al.^50^ reported that applying biochar produced from dairy manure to sandy loam soil increased the quantity of macroaggregates.

These diverse findings underscore the complexity of biochar’s influence on soil properties, which can depend on factors such as biochar type, application rate, soil type, pyrolysis temperature, and environmental conditions. From these factors, pyrolysis temperature plays a crucial role on its effects on soil aggregate stability. Different pyrolysis temperatures result in biochars with distinct physicochemical properties. Low-temperature pyrolysis yields biochar rich in volatile organic compounds, ash content, and higher yield and cation exchange capacity (CEC), while high-temperature pyrolysis produces biochar with increased carbon content, pH, electrical conductivity (EC) and high surface area^9,54^. These variations in biochar properties influence their interactions with soil particles and microorganisms, ultimately affecting soil aggregate stability. While low-temperature biochar may enhance soil aggregation by promoting microbial activity and organic matter accumulation, high-temperature biochar may contribute to soil stability through the formation of stable pore structures. However, optimal pyrolysis temperature may vary depending on soil type, climate conditions, and intended application. Future research should focus on elucidating the specific mechanisms underlying these effects and developing tailored biochar formulations for different soil management scenarios.

#### Effect of biochar on water repellency

**Figure 3:**
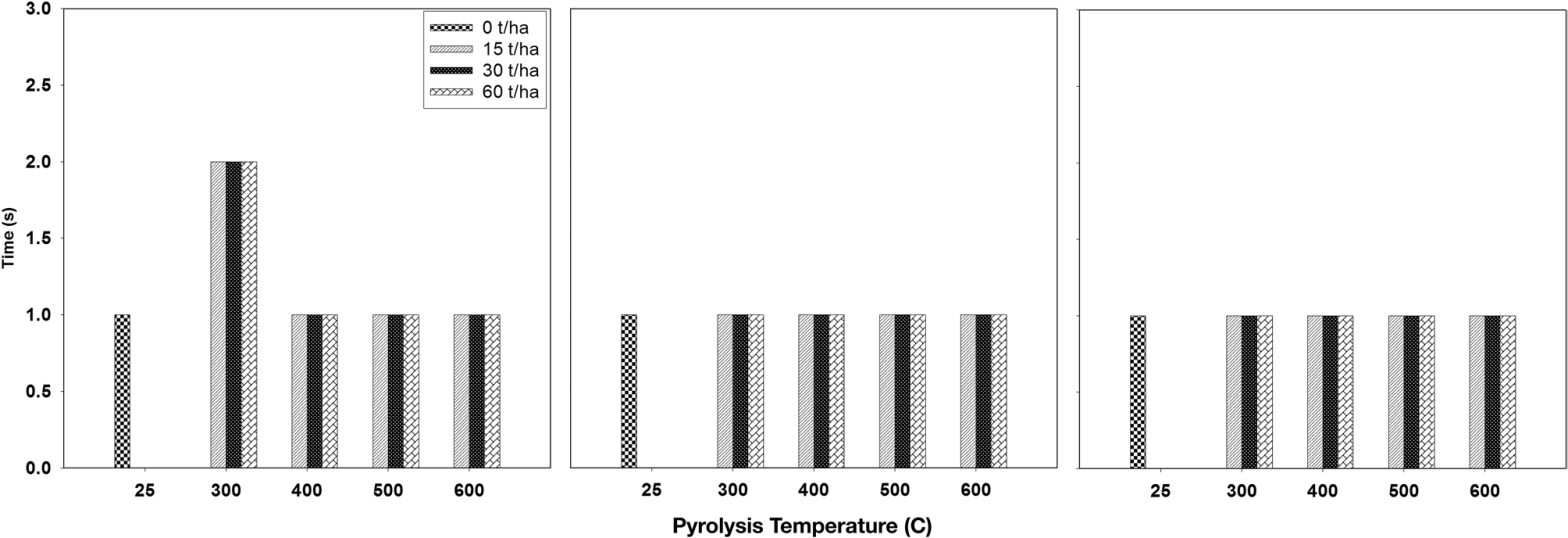
Effect of biochar that produced under different pyrolysis temperature and application rates after first (left), second (middle) and third (right) month of incubation on soil water repellency. Values are averages over three replicates of the treatments, with error bar representing the standard error on y-axis.

Figure 3 illustrates the outcomes of our study on the impact of biochar produced at various pyrolysis temperatures and applied to the soil at varying rates, following a 3-month incubation period, on water repellency. The data consistently revealed that applying biochar to the soil had no discernible effect on water repellency. This observation held true across all pyrolysis temperatures, application rates, and incubation durations. Our assessment using the Water Drop Penetration Time test (WDPT) consistently indicated that the maximum time required for a water droplet to infiltrate the soil surface was less than 5 s. This rapid infiltration time falls below the threshold level for repellency, signifying that the treated surface remained wettable and did not exhibit water-repellent characteristics.

Our findings align with prior research in this area, corroborating the consistent observation that biochar application does not significantly impact soil wettability. Abel et al.^55^ demonstrated that applying biochar produced from maize feedstock at 750 °C to sandy soil had no discernible effect on wettability. Similarly, Baronti et al.^56^ reported that applying biochar derived from orchard waste to the soil did not alter water repellency. Furthermore, Głąb et al.^57^ found that when biochar was applied to loamy sand soil, it had no notable effect on soil wettability. The Water Drop Penetration Times (WDPTs) were consistently less than 5 s in all the biochar treatments examined in their study, indicating that the treated soils remained non-water repellent. These collective findings underscore the consistent conclusion that biochar application, regardless of source material or pyrolysis temperature, tends to maintain or have minimal impact on soil wettability, keeping the treated soil surfaces in a wettable state.

#### Effect of biochar on penetration resistance

**Figure 4:**
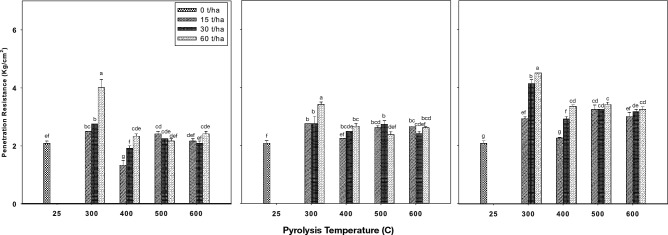
Effect of biochar that produced under different pyrolysis temperature and application rates after first (left), second (middle) and third (right) month of incubation on penetration resistance. Values are averages over three replicates of the treatments, with error bar representing the standard error on y-axis. Means within same incubation time (subfigure) followed by the same letter are not significantly different at the ⍶ = 0.05 probability level according to Tukey’s honestly significant difference (HSD) test.

Figure 4 presents the findings from our study, which examined the impact of biochar produced at different pyrolysis temperatures and applied to the soil at varying rates over a 3-month incubation period on soil penetration resistance. In the first month of incubation (Fig. 4a), the results indicated that penetration resistance exhibited different patterns depending on the pyrolysis temperature and application rate. Under 300 °C, 400 °C, and 600 °C pyrolysis temperatures, penetration resistance increased with the application rate, while it decreased under 500 °C. Specifically, at 300 °C, penetration resistance initially increased compared to the control and then decreased with rising pyrolysis temperatures. For instance, it decreased from 4 at 300 °C to 2.4 kg cm^−2^ at 600 °C after one month of incubation at an application rate of 60 tons per hectare. In the second month of incubation (Fig. 4b), the results demonstrated that penetration resistance increased with biochar application compared to the control. Under the conditions of the 2 months incubation and an application rate of 60 ton ha^−1^, penetration resistance increased to 3.4 kg cm^−2^ compared to 2.1 kg cm^−2^ for the control. After the third month of incubation (Fig. 4c), the findings revealed that at all pyrolysis temperatures, penetration resistance increased with the biochar application rate, reaching a maximum value of 4.5 kg cm^−2^ at 300 °C pyrolysis temperature and an application rate of 60 tons per hectare. Our results are in line with previous findings reported by Busscher et al.^58^, which demonstrated a similar trend in their study, applying biochar produced from pecan shells at 700 °C to loamy sand soil at a rate of 2.1% increased penetration resistance from 1.19 to 1.27 MPa after 44 days of incubation. The impact of biochar in a short period of time may not lead to changes in some soil properties. In other words, long periods of time are needed to observe the effect of biochar on the interaction between biochar and soil. However, this time period may not be the same for different soil properties. Blanco-Canqui^19^ by reviewing previous studies concluded that adding biochar to soil may not lead to a decrease in soil penetration resistance. This consistency in the observed increase in penetration resistance following biochar application strengthens the evidence for the impact of biochar on soil properties such as soil compaction and penetration resistance.

#### Effect of biochar on soil organic matter content

**Figure 5:**
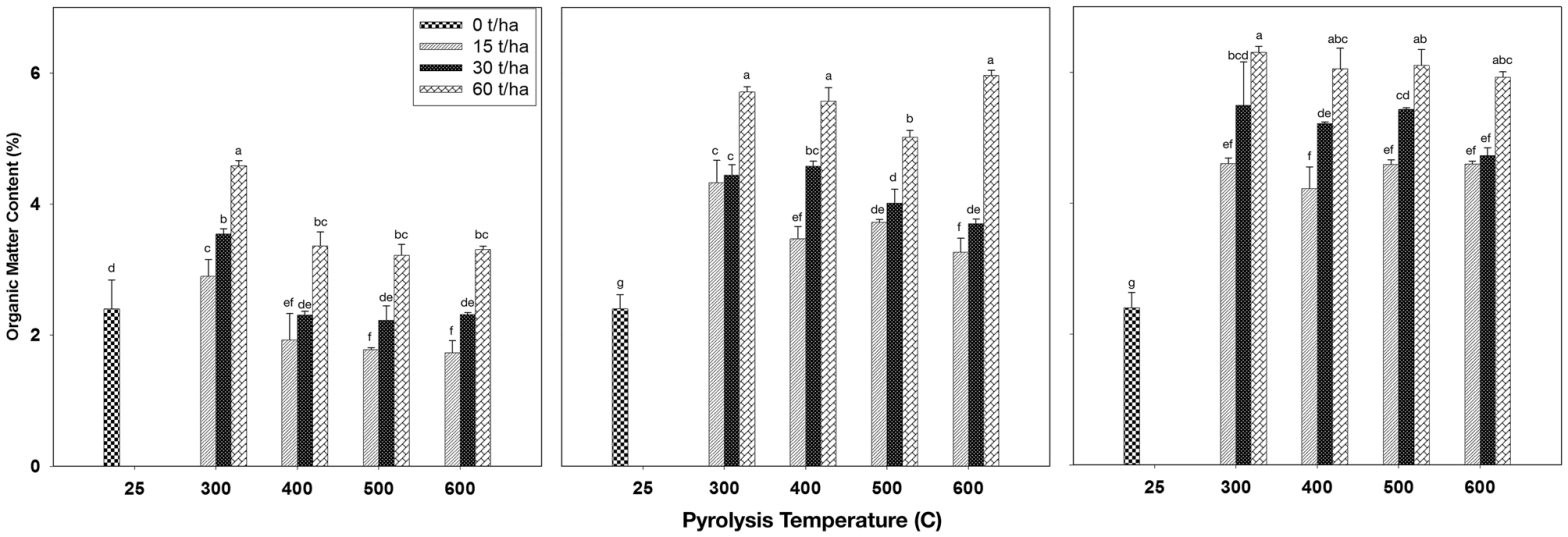
Effect of biochar that produced under different pyrolysis temperature and application rates after first (left), second (middle) and third (right) month of incubation on the percentage of organic matter content. Values are averages over three replicates of the treatments, with error bar representing the standard error on y-axis. Means within same incubation time (subfigure) followed by the same letter are not significantly different at the ⍶ = 0.05 probability level according to Tukey’s honestly significant difference (HSD) test.

Figure 5 illustrates the impact of biochar produced at varying pyrolysis temperatures and applied to the soil at different application rates after a three-month incubation period on organic matter content. The findings reveal that, in comparison to the control, soil organic matter content increased as the rate of biochar application increased after the first (Fig. 5a), second (Fig. 5b), and third (Fig. 5c) months of biochar application. For instance, in the treatment with a pyrolysis temperature of 300 °C and biochar application rates of 30- and 60-ton ha^−1^, organic matter (OM) content increased from 1.2% in the control to 2.68% and 4.04%, respectively. The highest value, 4.43%, was observed after the third month of incubation at a pyrolysis temperature of 300 °C and a biochar application rate of 60 ton ha^−1^. Analyzing the organic matter content over time, the results indicate varying trends. For example, at a pyrolysis temperature of 300 °C and an application rate of 30 ton ha^−1^, organic matter content increased from 2.6% after the first month of incubation to 3.5% after the third month. Conversely, at a pyrolysis temperature of 500 °C and an application rate of 15 ton ha^−1^, organic matter content decreased from 1.49% after the first month of incubation to 1.3% and 1.1% after the second and third months of incubation, respectively. These findings align with previous research by Zimmerman et al.^59^, which demonstrated that biochar’s surfaces and pore spaces adsorb soil organic matter, thereby conserving it from decomposition. Tang et al.^60^ demonstrated a significant increase in soil organic matter, reaching up to 121% compared to the control, as the biochar application rates were augmented. Ji et al.^61^ have shown that biochar exhibits a strong adsorption affinity for organic matter, potentially inhibiting organic carbon mineralization. Incorporating biochar into the soil at a rate of 7.7 g kg^−1^ reduced the mineralization of soil organic matter from 6.6% to 6.3%, as Bruun and EL-Zehery^62^. Furthermore, Bhattarai et al.^63^ found that applying biochar to the soil at a rate of 10 ton ha^−1^ increased organic matter content from 1.2% in the control to 2.2%.

#### Effect of biochar on soil pH

**Figure 6:**
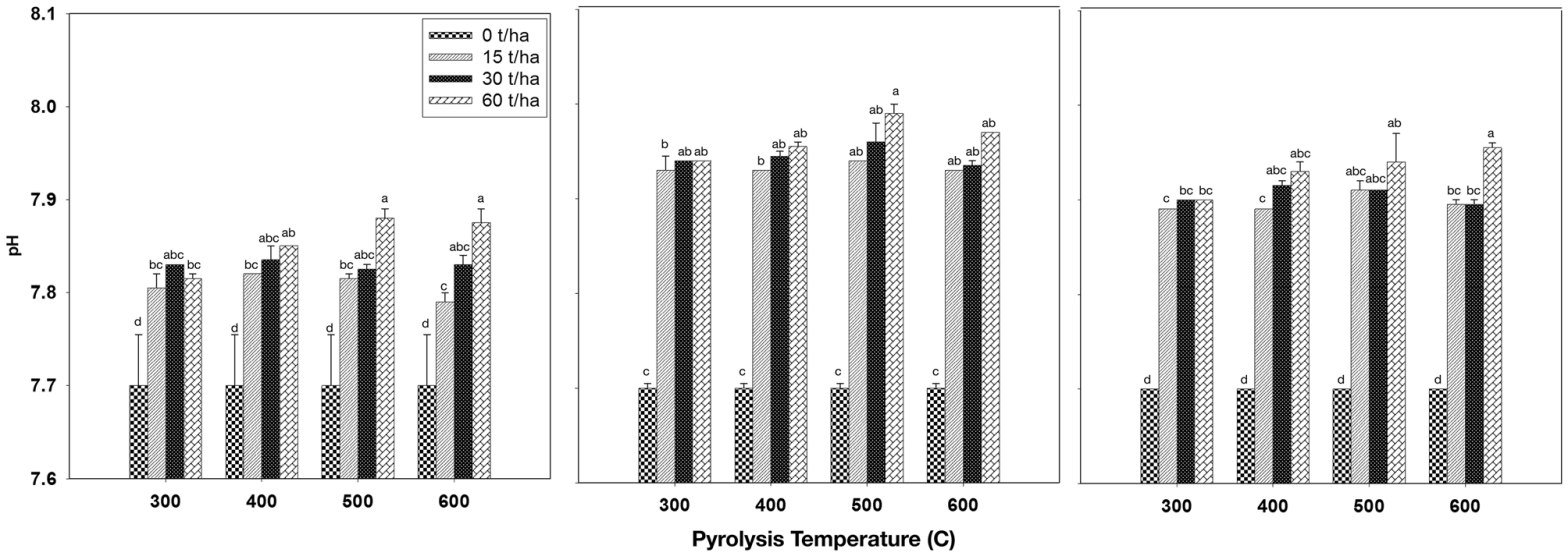
Effect of biochar that produced under different pyrolysis temperature and application rates after first (left), second (middle) and third (right) third month of incubation on the soil pH. Values are averages over three replicates of the treatments, with error bar representing the standard error on y-axis. Means within same incubation time (subfigure) followed by the same letter are not significantly different at the ⍶ = 0.05 probability level according to Tukey’s honestly significant difference (HSD) test.

Figure 6 displays the influence of biochar produced at various pyrolysis temperatures and applied at different application rates to the soil following a three-month incubation period, focusing on soil pH. The results consistently demonstrate an increase in soil pH following biochar application, as evidenced in the first (Fig. 6a), second (Fig. 6b), and third (Fig. 6c) months of incubation. The rise in soil pH is more noticeable as the biochar application rates increase compared to the control treatment, particularly during the first and second months. However, after the third month of incubation, when biochar was applied at a rate of 15 ton ha^−1^ at pyrolysis temperatures of 300 °C and 400 °C, it had no significant impact on soil pH. In contrast, at pyrolysis temperatures of 500 °C and 600 °C with the same application rate, there was a slight increase in soil pH compared to the control treatment. Moreover, soil pH demonstrated a correlation with increasing pyrolysis temperature. The highest pH value observed was 7.99 after the second month of incubation, achieved at a pyrolysis temperature of 500 °C and an application rate of 60 ton ha^−1^. Al-Wabel et al.^64^ stated that soil pH increases from 6.7 to 9.7 and 12.4 by increasing pyrolysis temperature of conocarpus biochar production from 200 to 400 and 800 °C. When examining the pH trend over the incubation period, the results indicate that soil pH increased notably after the second month of incubation, followed by a slight decrease after the third month of incubation. Changes in soil pH resulting from biochar application are closely tied to the pH of the biochar used. When the biochar pH exceeds that of the soil, it leads to an increase in soil pH, while if the biochar pH is lower than the soil pH, it causes a decrease in soil pH. These findings align with research conducted by Cheng et al.^65^, who demonstrated that applying biochar with a pH value of 5.83 to soil with a pH value of 4.33 resulted in an increased pH in the soil.

Additionally, Chintala et al.^66^ reported that the application of biochar to the soil at rates of 52, 104, and 156 ton ha^−1^ raised the pH from 4.78 in the control treatment to 5.51, 5.77, and 6.14, respectively, further supporting the correlation between biochar pH and soil pH changes. It's worth noting that the decrease in pH observed in the later stages of the incubation period can be attributed to the nitrification of NH_4_^+^ ions generated during the mineralization of organic nitrogen. This phenomenon has been documented by previous studies, including those by Yan et al.^67^ and Tang et al.^68^.

#### Effect of biochar on soil electrical conductivity

**Figure 7:**
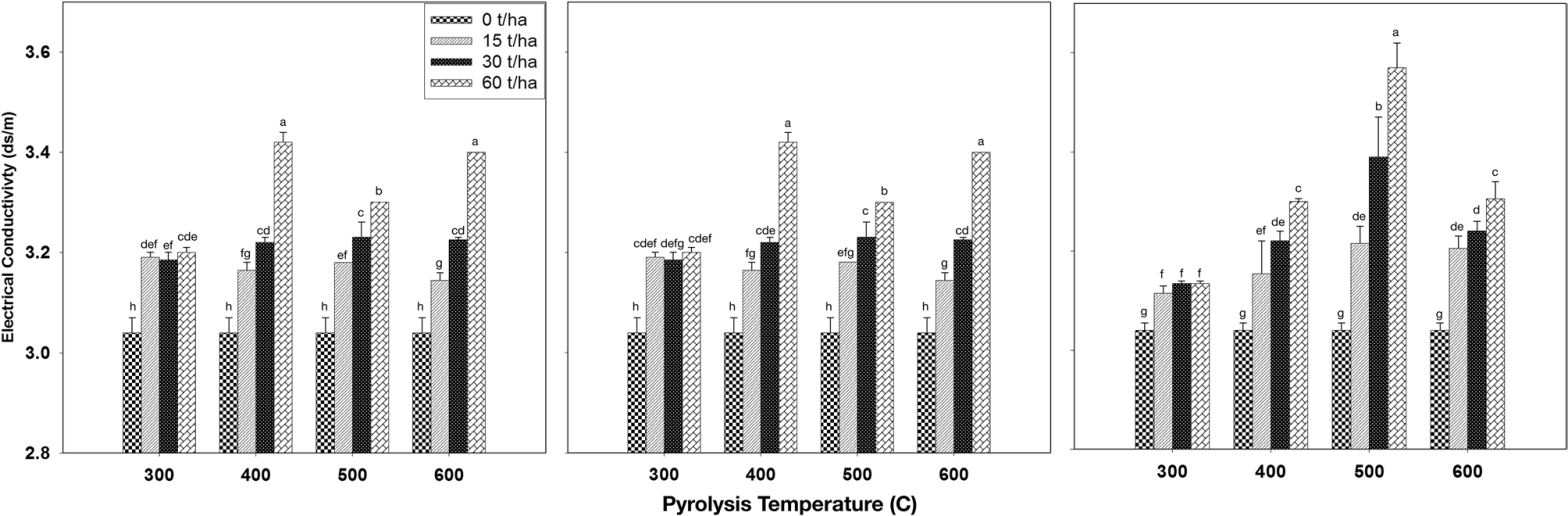
Effect of biochar that produced under different pyrolysis temperature and application rates after first (left), second (middle) and third (right) month of incubation on the soil electrical conductivity. Values are averages over three replicates of the treatments, with error bar representing the standard error on y-axis. Means within same incubation time (subfigure) followed by the same letter are not significantly different at the ⍶ = 0.05 probability level according to Tukey’s honestly significant difference (HSD) test.

Figure 7 presents the impact of biochar produced at different pyrolysis temperatures and applied at varying application rates to soil after a 3-month incubation period, focusing on soil electrical conductivity (EC). Across all incubation periods and pyrolysis temperatures, the results consistently indicate that soil electrical conductivity increased as the biochar application rate increased compared to the control treatment. The highest EC value recorded was 3.6 dS m^−1^ after the third month of incubation when biochar was applied at a rate of 60 ton ha^−1^ and produced at 500 °C. This increase in EC was particularly pronounced at the 60-ton ha^−1^ application rate in all months and at all pyrolysis temperatures.

Specifically, at 300 °C, with all application rates, the results show that electrical conductivity decreased over time during incubation. At 400 °C, there were no significant differences in electrical conductivity with respect to incubation time at application rates of 15 and 30 ton ha^−1^. However, at the 60 ton ha^−1^ rate, soil electrical conductivity decreased over the incubation period. Conversely, at 500 °C, with all application rates, the results indicate that electrical conductivity increased with increasing incubation time. However, at 600 °C, with a 15-ton ha^−1^ application rate, electrical conductivity increased with incubation time, while at 30 ton ha^−1^, it initially decreased in the second month but then increased in the third month. At the highest application rate of 60 tons per hectare at 600 °C, the results showed a decrease in electrical conductivity over the incubation period.

These results are in line with previous research findings, further substantiating the relationship between biochar application and soil electrical conductivity. The study conducted by Zhang et al.^69^ demonstrated that applying biochar produced from Cotton gin trash to the soil at a rate of 45 ton ha^−1^ led to an increase in the electrical conductivity of sandy loam soil. Similarly, Khaledi et al.^70^ reported a higher soil EC value after applying 5% of biochar compared to the control. Chintala et al.^66^ observed an increase in soil electrical conductivity when biochar was applied at rates of 52, 104, and 156 ton ha^−1^, with values rising from 0.09 dS m^−1^ to 0.11, 0.14, and 0.15 dS m^−1^, respectively. Abrishamkesh et al.^71^ reported a similar trend, showing that the application of biochar produced from rice husk to the soil at rates of 2.4% and 3.3% resulted in increased electrical conductivity, with values of 0.62 dS m^−1^ and 0.695 dS m^−1^, respectively, compared to 0.593 dS m^−1^ in the control treatment. The rise in soil electrical conductivity (EC) can be attributed to the elevated EC of the applied biochar. As the application rate increases, it implies the addition of more salts to the soil^9,54^, resulting in a higher soil EC^72^.

## Conclusion

In conclusion, this study underscores biochar’s significant potential in enhancing soil’s hydro-physical properties, thereby offering promising avenues for improving soil structure, water retention, and overall agricultural productivity. The investigation conducted on sandy loam soil amended with biochar derived from olive pomace (Jift) across varying pyrolysis temperatures and application rates revealed notable impacts on soil characteristics. Specifically, at a pyrolysis temperature of 300 °C, biochar incorporation led to increased aggregate stability, with the highest improvement observed after 60 days of incubation. Moreover, this pyrolysis temperature demonstrated a decreased infiltration rate and increased water retention, indicative of enhanced soil moisture management. However, contrasting effects were observed at other pyrolysis temperatures, suggesting the nuanced influence of biochar properties on soil dynamics. Overall, the findings highlight the beneficial effects of biochar on soil physical and hydraulic properties, with the 300 °C pyrolysis temperature showing the most favorable outcomes under agriculturally relevant conditions. Nonetheless, comprehensive field assessments are warranted to elucidate the long-term implications of biochar application on hydro-physical properties, thus providing invaluable insights for sustainable soil management practices.

## Data Availability

All data generated and analyzed during this study are included in this published article.
